# Flexural Behaviour and Internal Forces Redistribution in LWAC Double-Span Beams

**DOI:** 10.3390/ma14195614

**Published:** 2021-09-27

**Authors:** Ewelina Kołodziejczyk, Tomasz Waśniewski

**Affiliations:** Department of Concrete Structures, Lodz University of Technology, Politechniki 6, 93-590 Łódź, Poland; ewelina.kolodziejczyk@p.lodz.pl

**Keywords:** lightweight aggregate concrete, LWAC, ductility, bending, redistribution

## Abstract

This research study aimed to investigate the effect of the lightweight aggregate concrete and steel reinforcement interaction on the behaviour of continuous beams compared to the normal concrete of the same strength. This paper presents six full-scale, double-span beams with a rectangular cross-section made of both lightweight and normal concrete. The study confirmed that beams made of lightweight aggregate concrete achieve comparable flexural capacities to those made of NWC but their deformability and ductility are lower. Although the redistribution of internal forces depends mainly on the longitudinal reinforcement ratio, the influence of ultimate compressive strains of concrete is also noticeable. The ultimate compressive strains in LWAC are generally lower than in NWC. The lower rotational capacity of LWAC results in smaller degrees of moment redistribution in beams made of this concrete compared to normal concrete beams.

## 1. Introduction

Modern engineering is constantly looking for solutions to achieve the largest possible spaces without obstacles (columns and walls); however, as the span of slab or girder increases, the share of self-weight in the total sum of the loads becomes greater. This correlation strongly influences the final deflections of the structure. To reduce the self-weight of the structure, volumetric inserts can be used, such as Bubbledeck [1] or Cobiax [2] systems. The weight reduction resulting from the replacing of concrete with air or other lightweight materials can reach up to 35%. However, the use of such solutions is associated with technical difficulties.

Another way to reduce the self-weight of the structure, which is simpler and more reliable, is to use lightweight cementitious composites. The main principle of obtaining lightweight concrete is to replace the solid material with air voids. If air is contained in the aggregate, the final product is called lightweight aggregate concrete. If cement paste is replaced by air voids, the overall matrix is cellular concrete.

Lightweight aggregates can be of natural origins such as pumice [3], scoria, volcanic tuff, and lava ash. Other sources of lightweight aggregates can be mineral resources such as sintered shale and clay (LECA [4], LIAPOR [5], and expanded perlite [6,7]. Moreover, lightweight aggregate concrete can be made based on aggregates constituting a waste product instead of natural aggregates, such as power plant slag, sintered fly ash (CERTYD, LYTAG, and POLLYTAG [8]), granulated blast furnace slag, metallurgical pumice, and fly ash cenospheres [9,10,11]. In the era of diminishing natural resources, sustainable development and the search for alternative material solutions for buildings and structures, in particular, are becoming especially important. 

There are also attempts to use waste from other industries, such as PET bottles [12,13] or plastic (PVC) obtained from electronic waste [14,15]. Reusing waste materials from agricultural wastes is another way to produce lightweight cementitious composites. These so-called Green-LWAC [16] use materials such as oil palm shells [17,18], coconut shells [19,20], hemp flour [21], or jute fibres [22].

Recently, modern and innovative lightweight cementitious composites based on glass microspheres (HGMS) [23,24], nanoparticles (carbon nanotubes and nanofibers, and silica nanoparticles) [25,26,27], and aerogel [28] have also been developed.

These crucial features and a wide range of modern solutions encourage the use of lightweight concrete in structural engineering applications.

### 1.1. Research Significance

There is a general scepticism regarding the use of lightweight aggregate concrete (LWAC) for structural applications. This concern is attached its higher brittleness and lower tensile strength, shear resistance, and modulus of elasticity compared with normal-weight concrete (NWC). These characteristics can lead to a reduced ductility of reinforced concrete structures [29,30,31,32].

The ductility of reinforced concrete elements can be defined as the ability to plastically deform in the area of permissible loads and also above it. High ductility means that the structure can transfer loads despite the overloading of its critical sections. Admittedly, excessive displacements and deflections may occur, but the structure’s load capacity will be maintained.

It should be noted that most experimental investigations [33,34,35,36,37,38] regarding LWAC beams usually have focused on examining the flexural performance and ductility of statically determinate beams with simple supports. In most cases, however, reinforced concrete structures are designed as statically indeterminate systems, such as continuous beams, frames, or slabs. In these types of structures, adequate ductility ensures the ability to rotate critical cross-sections, allowing for the proper redistribution of the internal forces.

Most of the tests for statically indeterminate structures involve members made of normal-weight concrete. There is still little data on the behaviour of continuous elements made of lightweight concrete and its influence on both the development of plastic hinges and the redistribution of internal forces. Moreover, LWAC is made based on aggregates with various properties that strongly determine the final characteristic of this type of concrete.

### 1.2. Lightweight Aggregate Concrete Characteristic According to Eurocode 2

In Eurocode 2 [39], density was adopted as the main parameter for classifying lightweight aggregate concrete. For each class and compressive strength, the material parameters such as modulus of elasticity or ultimate compressive strains are obtained from parameters of the corresponding regular weight concrete by multiplying them by the coefficient based on the oven-dry density of the LWAC:

(1) $$\eta_{E}={\left(\frac{\rho }{2200}\right)}^{2}$$

where *ρ* is the oven-dry density for the relevant class of the LWAC.

This approach results in a large diversity of the possible LWAC classes with different characteristics.

**Figure 1:**
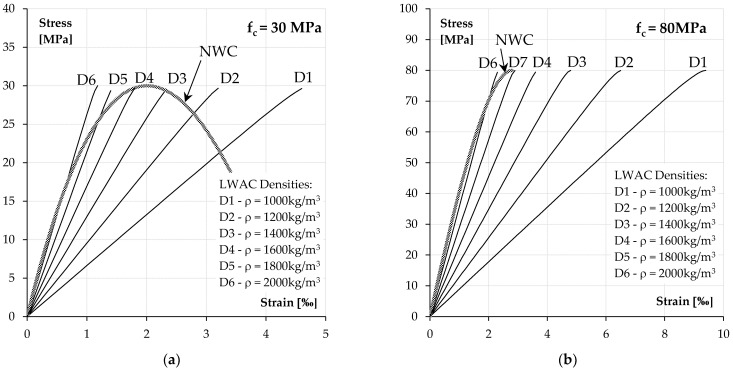
Stress-strain characteristics of LWAC and NWC concrete for two selected strength classes: (**a**) *f_c_* = 30 MPa and (**b**) *f_c_* = 80 MPa.

For example, Figure 1 shows the strength characteristics determined according to [39] for the two selected classes of lightweight concrete and normal concrete of the same strength. As we can see, all the characteristics of lightweight concrete are highly linear compared to normal concrete. Moreover, lightweight concrete characteristics have a very steep post-peak softening branch. For this reason, Eurocode 2 [39] require the rejection of this part of the characteristic and assume that the strains at the maximum stress level are equal to the ultimate. This is similar to the behaviour of the high-strength normal-weight concrete.

It should also be noted that the standard recommendations for determining the permissible redistribution of bending moments are empirically determined using the data and observations of the elements made of normal concrete [40]. The reliability of these recommendations in determining the moment redistribution capacity of continuous LWAC beams is still unclear because these beams commonly exhibit a lower displacement ductility ratio than NWC beams with the same longitudinal reinforcement [32].

The following sections will present our experimental investigations, results of the beam tests, and analysis of the results in terms of the bearing capacity, ductility, and redistribution of internal moments. The last section presents the conclusions of this study.

## 2. Experimental Investigations

### 2.1. Test Programme

The test programme included six double-span beams with a rectangular cross-section. Three beams were made of lightweight concrete and the remaining three were made of normal concrete. The beams were divided into three groups differing in the reinforcement ratios over the middle support. The beams were designed according to Eurocode 2 [39]. The differences result from the selection of reinforcements, which was done in such a way so as to force various types of redistribution flows. The reinforcement system in the first group (BL1 and BN1) can be defined as close to elastic-consistent, i.e., that the load capacity of the support section will be achieved simultaneously with the load capacity of the span sections. In the second group of beams (BL2 and BN2), the redistribution of the bending moments from the support to the spans was forced (under-reinforced middle support cross-section). In the third group of beams (BL3 and BN3), the reinforcement was selected to redistribute the bending moments from the spans to the middle support (over-reinforced middle support cross-section).

### 2.2. Geometry and Reinforcement

**Figure 2:**
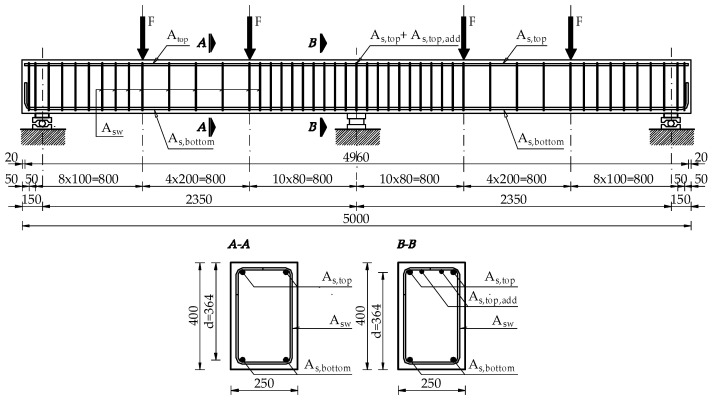
Details of specimen geometry and arrangement of reinforcement. Note: all dimensions are in mm.

Figure 2 shows the section geometry and reinforcement details of the beams. All specimens had the same nominal dimensions as follows: the width b and height h of the rectangular section were 250 and 400 mm, respectively; the length *L_span_* of a span measured between the centres of the exterior and middle support was 2350 mm; and the full-length *L_tot_* measured between the centres of both exterior supports was 4700 mm.

To serve as the basic longitudinal reinforcement of the beams placed along the entire length of the beam, two bars with a diameter of 16 mm each were assumed, placed both at the top and bottom of the section. The additional reinforcement above the middle support was made of 12 mm or 20 mm diameter bars. In order to protect the beams from shear failure in the support zones, the beams were reinforced with stirrups made of 8 mm diameter bars, spaced at 80 and 100 mm intervals. Table 1 presents the details of the beam specimens.

### 2.3. Materials

Table 2 summarizes the mechanical properties of the steel reinforcing bars measured from the three standard test specimens. All reinforcing bars exhibited clear yield plateau and strain-hardening characteristics, indicating similar ranges for the modulus of elasticity corresponding to approximately 200 GPa. The yield strength of the bars used for the longitudinal reinforcement ranged between 595.0 and 663.0 MPa.

**Figure 3:**
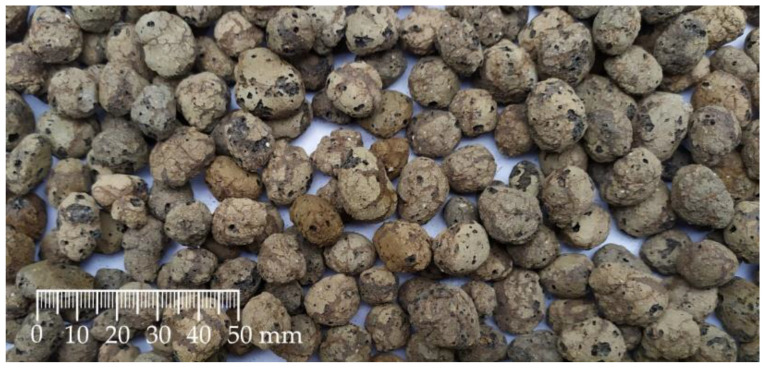
Certyd: a sintered ceramic lightweight aggregate.

Lightweight aggregate concrete based on a lightweight aggregate called Certyd [41] was selected for the construction of the beams. Certyd (Figure 3) is a sintered, ceramic, and porous aggregate. It was made according to the LSA (Lightweight Sintered Aggregate) technology, distinguished by an innovative method of conducting the sintering process in a rotary kiln. The main raw material used in the production of Certyd is fly ash from electrostatic precipitators and from an ash and slag mixture from wet furnace waste removal that was produced in the process of burning hard coal in the thermal–electric power station. Normal concrete was selected in such a way that its compressive strength corresponded most closely to the compressive strength of the lightweight aggregate concrete. Table 3 summarizes the mixture proportions of both types of concrete.

All beams were cast from the same batch of ready-mixed concrete. At the same time, standard samples were taken from the batch to test the strength properties of concrete; specifically, cylindrical samples were taken to determine the compressive strength *f_c_*, *f_lc_* and modulus of elasticity *E_c_,E_lc,_*, as well as the sigma-epsilon characteristic. Compressive strength *f_c,cube_, f_lc,cube_* and splitting tensile strength *f_ct,sp_, f_lct,sp_* were determined on cubic samples. The age of the concrete during the tests was in the range of 152 ÷ 164 days. The LWAC and NWC properties determined on the test day are shown in Table 4.

### 2.4. Test Setup and Instrumentation

**Figure 4:**
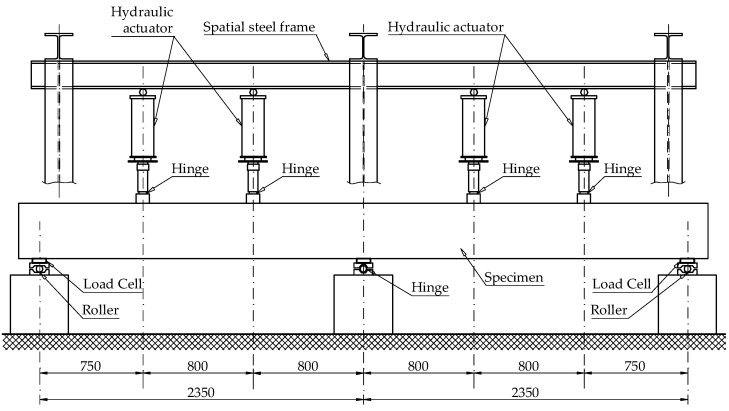
Test setup of the double span beams.

Figure 4 illustrates the test setup. The beams were tested in a seven-point bending configuration, with three supports distanced 2350 mm apart from each other and two loads applied at each span spaced at 800 mm apart from each other.

The two exterior supports were designed to allow for horizontal and rotational movement, whereas the intermediate support only allowed for rotation. To be able to determine the redistribution of the internal forces, the reactions at both extreme supports were recorded using load cells.

**Figure 5:**
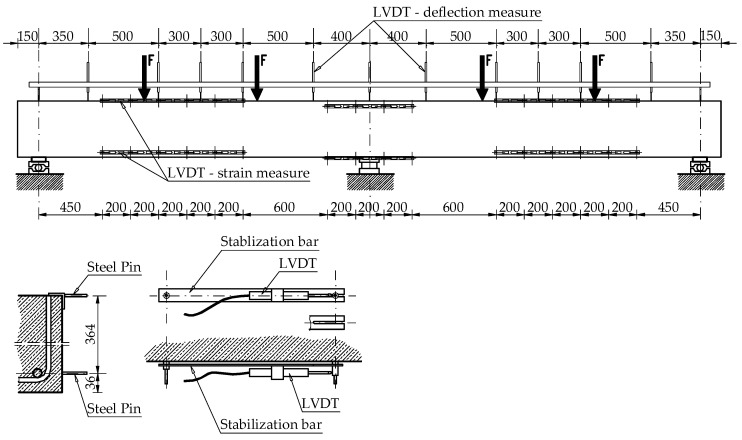
Specimen instrumentation scheme.

The instrumentation for each beam consisted of a set of linear variable differential transformers to measure the strain in the top and bottom fibres of the support and span sections. The measurement base was fixed at 200 mm. In addition, the deflections of both spans were also measured using a set of LVDT mounted on a special bar. The data were collected using a special computer data acquisition system. The instrumentation scheme is shown in Figure 5.

In addition, the data in the zones of expected plastic hinges were collected using an Aramis optical photogrammetry system.

## 3. Test Results

The summary of the test results is presented in Table 5. It should be noted that the first cracks appeared in the phase in which the load was increased by a step of 5 kN. Observations of cracks were conducted after the stabilization of the force at a given level.

In BN1 and BL1 beams, which were designed according to the linear bending moment distribution, the first flexural crack appeared at a maximum bending moment section in the span, almost at the same load level, namely at 35 kN in BN1 and 40 kN in BL1. In both cases, soon after, it was followed by a vertical crack at the intermediate support (50 kN in BN1 and 45 kN in BL1). In the second pair of beams with symmetrical longitudinal reinforcement, cracks in BN2 in the mid-span and at the support were recorded at the same load value of 50 kN. In BL2, cracks were noticed first at the intermediate support at 30 kN and then at the mid-span at 40 kN. In beams with the strongest top reinforcement, the first cracks appeared in the mid-span (25 kN in BN3 and 35 kN in BL3) and then at similar force for both beams at the support (50 kN in BN3 and 45 kN in BL3). In each pair, the first intermediate support crack was recorded earlier in the LWAC beam.

It should be noted that in the two-span scheme, the maximum bending moment occurs over the internal support. In the test, slightly higher flexibility of this support (elastic pad) disturbed the moment distribution in the initial phase of the test, underestimating the support moment and, as a result, the first cracks almost always appeared in the span. Later in the test, the influence of this phenomenon disappeared and during the reinforcement yielding phase, the behaviour of the beams was as expected.

The yielding state in the top and bottom reinforcement (Table 5) was determined according to LVDT readings. The transducers located above the intermediate support and under the force near the exterior support were considered. The base length in both cases was 200 mm.

As expected for the first pair of beams (BN1 and BL1), yielding of the top support reinforcement and the bottom reinforcement in the span occurred at similar force values. In the second case, in beams with symmetrical reinforcement arrangement, yield stress was reached first at the intermediate support, regardless of the type of concrete. In contrast, in beams BN3 and BL3 with over-reinforced support, the bottom span reinforcement yielded earlier. Besides the behaviour, the values of the recorded load level at the yielding point were also very close for each of the tested pairs of specimens.

### 3.1. Crack Pattern and Failure Modes

**Figure 6:**
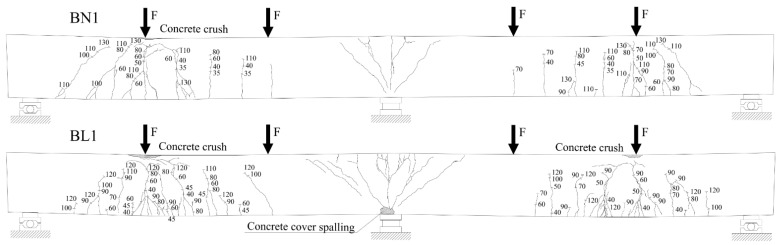
Crack propagation in BN1 and BL1 beams. Note: numerals given in figures indicate the applied load F (in kN). The drawings present the deformed beams after the removal of the load.

**Figure 7:**
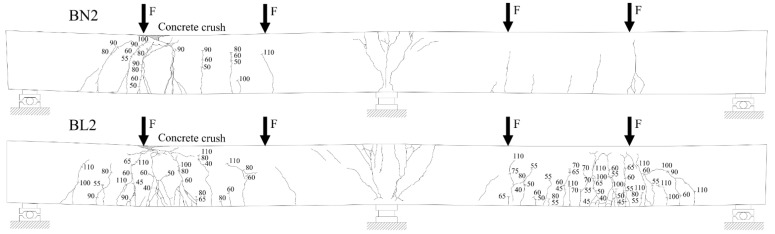
Crack propagation in BN2 and BL2 beams. Note: numerals given in figures indicate the applied load F (in kN). The drawings present the deformed beams after the removal of the load.

**Figure 8:**
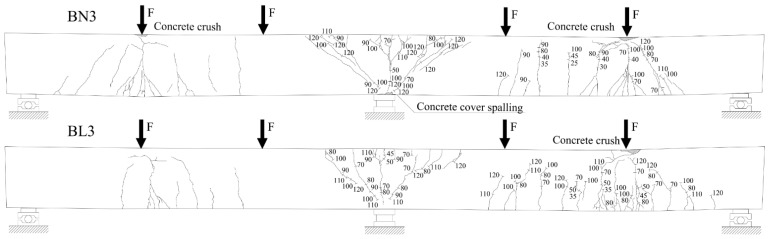
Crack propagation in BN3 and BL3 beams. Note: numerals given in figures indicate the applied load F (in kN). The drawings present the deformed beams after the removal of the load.

The crack propagation observed in the tested beams up to failure is presented in Figure 6, Figure 7 and Figure 8. Each figure shows a comparison of the two beams with the same reinforcement arrangement. The drawings also include the values of the load recorded during the crack monitoring. The areas without the numerals were covered by the Aramis system measurement, thus the observation of them could not be conducted during the test. In these places, only the wider cracks visible after the removal of the load were marked.

In all three figures, regardless of the type of reinforcement, it can be seen that there are differences in the cracking pattern of lightweight concrete beams and normal concrete beams. Cracks spacing in BL beams were smaller and the number of cracks were greater. This phenomenon is particularly visible at the bottom reinforcement level in the failed spans. The same tendency was observed in [35]. The reason for this is that the different fracture mechanism of both concretes, as in LWAC, results in the spreading of cracks to be almost unobstructed in the matrix, whereas in normal-weight concrete, natural course aggregate offers much higher resistance to their propagation [42]. It can be noticed that in BN beams in the mid-span, in the zone close to the pure bending, the main cracks were approximately vertical and their location corresponded to the arrangement of stirrups. In BL beams, cracking was more irregular and cracks were inclined towards the exterior force.

**Figure 9:**
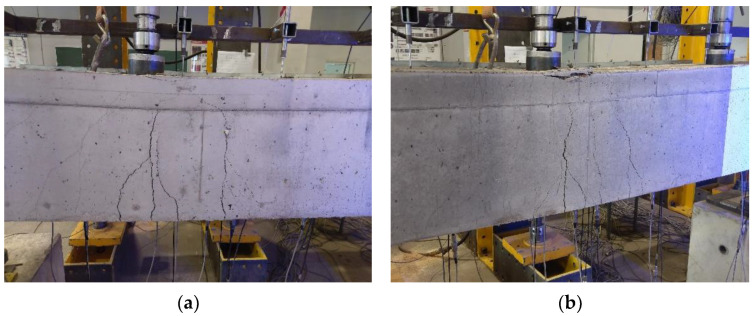
Failure observed at the mid-span zone in BN1 beam (**a**) and BL1 beam (**b**).

**Figure 10:**
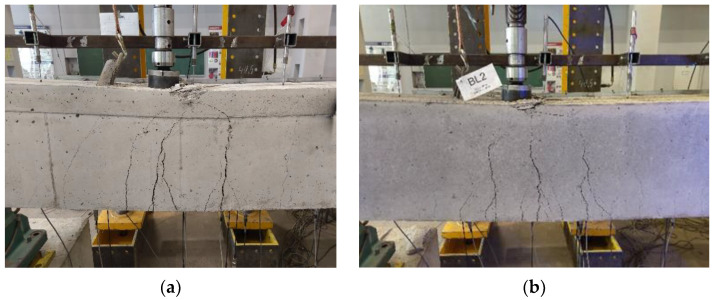
Failure observed at the mid-span zone in BN2 beam (**a**) and BL2 beam (**b**).

**Figure 11:**
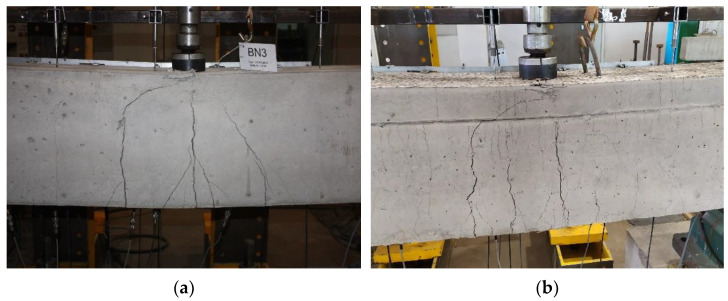
Failure observed at the mid-span zone in BN3 beam (**a**) and BL3 beam (**b**).

All tested beams failed with the crushing of concrete in the compression zone in the mid-span section. In all of the cases, it was preceded by yielding of the top and bottom reinforcement. The photos showing the failure state of the beams are presented in Figure 9, Figure 10 and Figure 11, and the values of the ultimate load and strain recorded in the critical sections at the ultimate state are given in Table 6.

It can be noticed that beams with the same reinforcement reached almost the same ultimate load, regardless of the type of concrete (see Table 6). As expected, the load-bearing capacity was higher the stronger the top reinforcement was, used at the support. The values of the strain of the tensile steel and concrete in the compression zone recorded with LVDTs were, in general, slightly higher for the beams made with NWC. The compressive strain of the extreme concrete fibre for NWC ranged between 2.2‰ and 9.0‰, and between 1.2‰ and 4.0‰ for LWAC. The main reinforcement reached the strain from 11.9‰ to 45.2‰ in the BN beams and from 9.0‰ to 28.9‰ in the BL specimens.

It is worth adding that strain records obtained with the traditional LVDT measurement and photogrammetric observation (Aramis) were consistent, but in both cases, especially concerning the reinforcement strain, they were affected by the specific location of the cracks, which was different in the two beams of each pair.

### 3.2. Load–Deflection Relationship

**Figure 12:**
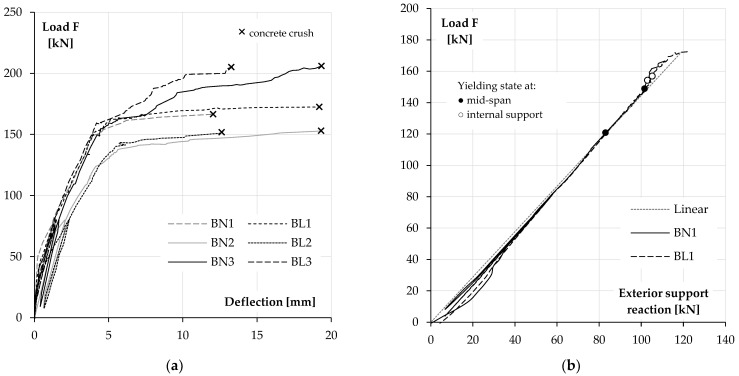
Load–deflection behaviour on NWC and LWAC beams (**a**). Load redistribution at exterior support in the first group (BN1 and BL1) (**b**).

Figure 12a presents the averaged mid-span deflections in different elements against the applied load. In all beams, we could observe the decrease of flexural stiffness after the initial cracks occurred. Another characteristic stage was the state of yielding in the reinforcement, revealed by a significant increase in the deflection at a small increment of the applied load. First, it appeared in the beams BN2 and BL2, and the process started in both beams at the force of about 120 kN with yielding of the top support reinforcement. Soon after, at about 138 kN, the yield state was reached in the mid-span. In the beam BL1, the yielding state was reached in both critical sections at a similar load value, namely 151.2 kN in the span section and 156.5 kN at the intermediate support. In BN1, the bottom reinforcement in the span yielded earlier but the result is uncertain (see Table 5). At the intermediate support, the top reinforcement reached the yield strength at a similar load value to the previous beam (154.1 kN). For these two pairs, there was a very slight increase in the recorded load values after the reinforcement reached the yield strength.

The third series of beams performed differently. In this case, first, the bottom reinforcement yielded (at 129.3 kN in BN3 and 139.5 kN in BL3), then the top support reinforcement yielded in both beams at a load of about 155 kN. This was followed by a significant increase in the load to about 210 kN in both beams until the concrete was crushed.

In beams designed according to elastic moment distribution (BL1 and BN1) higher ultimate deflection was reached in the LWAC specimen, but in the two other pairs, the mid-span displacement recorded for the beams made with NWC was bigger. The values of deflection are given in Table 7.

### 3.3. Support Reactions and Moment Redistribution

**Figure 13:**
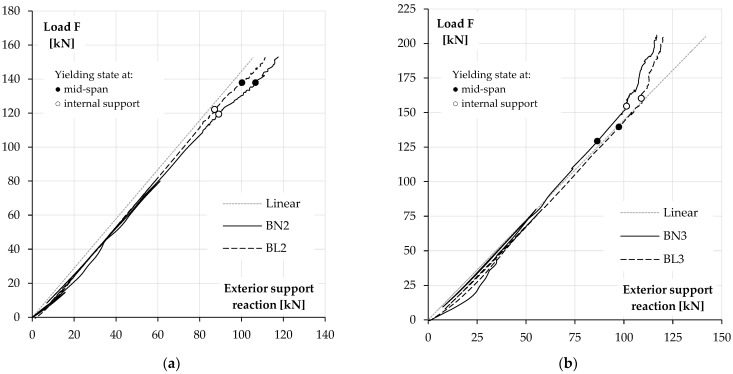
Load redistribution at exterior support in the second group (BN2 and BL2) (**a**) and the third group (BN3 and BL3) (**b**).

Redistribution of the internal forces in the tested beams is presented in Figure 12b and Figure 13. The diagrams show the applied load value versus the exterior support reaction for each pair of elements. The values corresponding to the yield points of the top and bottom reinforcement were also depicted in the figures.

The first stage of the tests was disturbed by the elastic pads, which made the intermediate support relatively more flexible. Their use caused an accidental redistribution of the load towards the exterior supports. The effect was stronger in BN beams due to their higher flexural stiffness. However, the disturbances disappeared at forces above 50 kN. For higher load, results obtained for the first and third group of beams were at first very similar to the elastic response, whereas in the second group, redistribution caused by stiffness degradations due to concrete cracking was slightly visible at the intermediate supports.

In the continuation of the test, the elements of all the series behaved as expected. In BL1 and BN1 beams, the distribution of the internal forces was elastic-compliant almost to failure, which was a consequence of the simultaneous degradation of the stiffness of the span and intermediate support sections. In both beams, a little later, yielding of the support reinforcement was observed and crushing of concrete in the span occurred shortly after that.

The reinforcement of the second group of beams (BN2 and BL2) was designed to obtain the redistribution of forces from the support to the spans, and in both cases, this aim was achieved. The diagrams show an increase in the reactions at the exterior supports in relation to the values from the elastic analysis as the load rises. It can also be observed that this process was accelerated when the support reinforcement reached the yield strain.

In the third case (BN3 and BL3), the highest reinforcement ratio was applied at the intermediate support. In both beams, the yield strain was first reached by the bottom reinforcement in the span. Soon after, a significant redistribution of the load to the internal support was initiated and continued until the failure of the elements.

Table 7 presents the degree of moment redistribution, namely *η* (%), calculated according to the method in [39] for the tested elements. The degree was determined for the records obtained at failure in the place of an expected plastic hinge, according to the formula:

(2) η = (1 − δ)⋅100,

where the coefficient of redistribution *δ* is defined as:

(3) $$\delta =\frac{M_{red}}{M_{elast}}$$

where *M_red_* is the ultimate bending moment calculated according to the averaged reaction value recorded in the test and *M_elast_* is the corresponding value from the elastic analysis.

The results presented in Table 7 indicate that the redistribution ability of LWAC sections in continuously reinforced elements is possible but is lower than in beams made with NWC. In the second group of beams where a simply supported failure mechanism was expected, creating of a plastic hinge at the intermediate support, the load redistribution ratio in the beam BN2 was 23.45% and only 11.33% in BL2, thus the ratio η was about twice as high for NWC. Similar results were obtained for the third group (double cantilever failure mechanism), where it was planned to achieve redistribution towards the intermediate supports and plastic hinge regions in the spans. In this case, the beam BN3 reached the load redistribution ratio of 18.09%, whereas BL3 reached 14.92%, notably about 18% lower. Table 7 also shows the corresponding ratios η for the sections in which an increase in the bending moment was obtained compared to the elastic analysis results. In BN1 and BL1, the values of the redistribution ratio were small, as expected. The values of the moment redistribution ratio calculated for the tested beams were compared with values predicted using Eurocode 2 [39] and ACI 318-14 [43].

According to [39], the redistribution of bending moments may be carried out without an explicit check on the rotation capacity provided that:

(4) $$\delta \;\geq \;0.44+1.25\left(0.6+\frac{0.0014}{\varepsilon_{cu2}}\right)\frac{x_{u}}{d}$$

for concrete strength lower than 50 MPa.

Where ε_cu2_ is the ultimate concrete strain, *x_u_* is the depth of the neutral axis at the ultimate limit state after redistribution, and *d* is the effective depth of the section. In addition,

(5) δ ≥ 0.7

when Class B or C reinforcement is used. The condition of Equation (3) relating to concrete was considered assuming *ε_cu2_* = 3.5‰ for NWC and *ε_lcu2_ = ε_cu2_* (0.4 + 0.6*ρ*/2200) for LWAC. In this analysis, its oven-dry density *ρ* was taken as 1840 kg/m^3^ and thus the ultimate limit strain of 3.16‰ was obtained.

The provisions given in [43] limit the decreasing bending moments in the critical sections by not more than 1000*ε_t_* %, with a maximum of 20%. Longitudinal tensile reinforcement strain should not be lower than 7.5‰ at the section at which moment is reduced.

**Figure 14:**
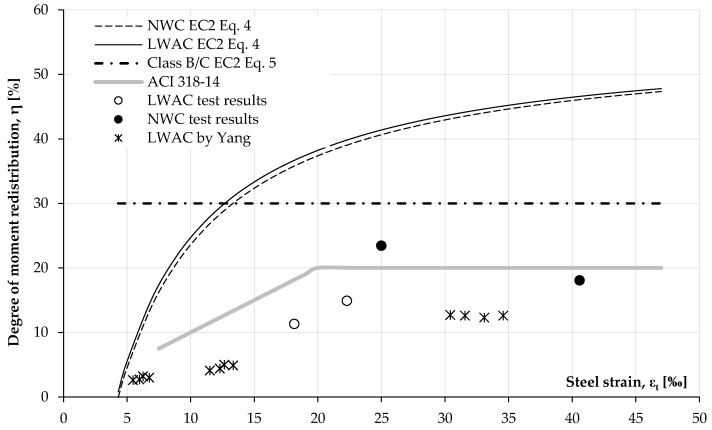
Comparison between the degree of moment redistribution values calculated according to test results (own tests presented below and elements tested by Yang [40]) and those predicted by both Eurocode 2 [39] and ACI 318-105 [43] equations.

Given these conditions, the predicted range of the degree of moment redistribution *η* shown in Figure 14 as a function of tensile reinforcement strain was obtained.

It can be noticed (Figure 14) that the predicted degree of moment redistribution *η* calculated according to the method in [39] reaches the maximum value for the tensile reinforcement strain at about 13‰ and then remains constant at the value of 30%. When it comes to the method in [43], the value varied linearly from 7.5% to the maximum permissible 20%.

The test results obtained in this study are also depicted in the diagram, separately for LWAC and NWC. The beams in the first group that performed elastic-compliant behaviour have been omitted. In both other cases, regardless of the reinforcement arrangement, a greater redistribution of forces was obtained in beams made of NWC.

Additionally, data were included from the research of 12 LWAC double-span beams as presented in [40]. In this case, only LWAC was considered. Concrete compressive strength was between 27.0 and 40.0 MPa. The beams were divided into three groups with different reinforcement ratios corresponding to the minimum, medium, and maximum reinforcement according to that in [43] (the medium reinforcement ratio is defined as an average of the minimum and maximum values). These tests showed that the degree of redistribution and ductility increased with the decreasing reinforcement ratio, which was correlated with greater strain in the tensile steel.

The results of BL2 and BL3 beams revealed to be slightly higher compared to the trend obtained in [40]. In all cases, however, the degree of moment redistribution in LWAC concrete elements was lower than that of NWC beams.

Moreover, it can be seen that both codes overestimate the degree of moment redistribution in LWAC beams but the difference is much more significant concerning the comparison with the values calculated according to the method in [39], which were much higher. In this case, NWC results were also lower than the predicted values using that in [39] but they showed much higher agreement with the predictions in [43].

### 3.4. Ductility Ratio

In order to determine the ductility of the tested beams, the displacement ductility ratio *µ*_Δ_ was calculated:

(6) $$\mu_{∆}=\frac{{∆}_{u}}{{∆}_{y}}$$

where Δ*_u_* is the average deflection of two spans of the beam at its ultimate state and Δ*_y_* is the average deflection at the load corresponding to the yield state in the section where we expected to observe a plastic hinge. The results are presented in Table 7.

The comparison of the results of the first group of beams, no matter which yield point of the reinforcement in the span was used for BN1, demonstrated that the BL1 beam (*µ*_Δ_ = 4.78) proved to be more ductile than the BN1 beam (*µ*_Δ_ = 4.14; see note 2 in Table 7). A different situation was observed for the two other pairs, where in both cases, elements made of NWC showed higher ductility and their ductility ratios were 5.03 (BN2) and 5.71 (BN3). For the beams made of LWAC, values of 3.01 (BL2) and 3.90 (BL3) were obtained.

### 3.5. Ultimate Load Capacity

**Figure 15:**
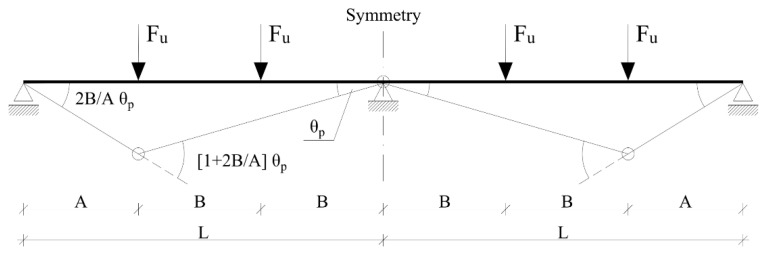
The collapse mechanism of the tested beams.

The ultimate load of the tested beams was determined according to the principle of complementary virtual work. The collapse mechanism of the element is presented in Figure 15. Typically, for a double-span beam, plastic hinges were assumed to occur in the points of maximum positive and negative bending moments.

For the presented scheme, the ultimate load F_u_ can be expressed as follows:

(7) $$F_{u}=\frac{\left(L/A\right)M_{Rd}^{+}+M_{Rd}^{-}}{3B}$$

where *M_Rd_^+^* and *M_Rd_^−^* are the load-bearing capacities of the doubly reinforced cross-sections calculated for the span and intermediate support, respectively. *A*, *B*, and *L* are the distances and they are presented in Figure 15. The load-bearing capacities were calculated according to the method in [39]. The rectangular stress diagram was assumed in relation to the concrete compression zone. The reinforcing steel stress in the tension and compression zones was calculated according to the elastic-perfectly plastic model. Experimental values of the compressive strength, yield strength, and modulus of elasticity were assumed. The results of this analysis are presented in Table 8.

Experimental values of the ultimate load recorded in the test are included in Table 8 and compared with the values calculated according to the method in [39]. In all cases, we obtained a very good agreement between the measured and calculated ultimate load values. Only in one beam (BN1), the result recorded in the test was slightly lower than the predicted value.

## 4. Conclusions

The pair of beams BL1 and BN1 should be excluded from the following conclusions. The elastic-compliant arrangement of the longitudinal reinforcement in these beams causes their high sensitivity to asymmetric loading and local load effects (local concrete crushing), which are very difficult to avoid in statically indeterminate setups. The authors believe that for this reason, the premature failure of beam BN1 occurred. This is confirmed by the calculations of the ultimate load capacity of this beam, which indicate a lower experimental load capacity than the calculated one (see Table 8).

The main findings and conclusions obtained from this study are the following:

- All tested beams failed with the crushing of concrete in the compression zone in the mid-span section after the yielding of the reinforcement steel in this section. In beams with forced redistribution of bending moments, the ultimate flexural capacities are essentially the same in each pair, regardless of the concrete used, and the effect of compressive strength is negligible. The experimental loads for all beams are in very good agreement with the load-bearing capacity values calculated according to the method in [39]. The mean and standard deviation of the relation of the experimental and calculated values of the load are 1.03 and 0.01 for NWC beams (BN2 and BN3), and 1.02 and 0.01 for LWAC elements (BL2 and BL3). Thus, the nominal moment capacity based on the equivalent stress block model [39] coincides with the flexural strength of the LWAC beams, which is the same as for NWC beams.
- The crack morphology in the beams made of LWAC differs from that of the beams made of normal concrete. Cracks spacing in BL beams was smaller and the number of cracks was greater. The reason for this is the different fracture mechanism of both concretes as in LWAC and the spreading of cracks was almost unobstructed in the matrix, whereas in normal-weight concrete, natural course aggregate offers much higher resistance to their propagation.
- During the test, both the lightweight concrete beams and the normal concrete beams somehow showed ductile behaviour but clear differences in the results can be seen. In beams with a reinforcement system forcing significant redistribution, the yield deflections were always smaller in the beams made of normal concrete than in the beams made of LWAC, this is evident considering lightweight aggregate concrete has a lower modulus of elasticity. However, the ultimate deflections were consistently larger in beams made of normal concrete, indicating the greater ductility of sections made of normal concrete. This conclusion is confirmed by displacement ductility ratio values. In the second group, in which redistribution from the internal support to the spans was planned, the ratio was 5.03 for the NWC beam and 3.01 for the LWAC specimen, thus about 40% less. Similarly, in the third group, with the over-reinforced intermediate support, the ductility ratio was 5.71 and 3.90 for NWC and LWAC elements, respectively. Thus, the difference is about 30%. These significant differences in ductility were reflected also in the redistribution results.
- A redistribution of moments occurred in all the tested beams from groups two and three in a manner consistent with expectations. However, there was an influence of the concrete type on the load redistribution ratio. Although the redistribution of internal forces mainly depended on the longitudinal reinforcement ratio, the influence of the ultimate compressive strains of concrete was also noticeable; the ultimate compressive strains in LWAC were generally lower than those in NWC, which also means lower strain in the reinforcement at the ultimate stage as well as a lesser ability to redistribute the load before the crushing of concrete occurs. This effect is evident regardless of the direction of force redistribution. In the tested beams, the degree of moment redistribution was lower for beams with LWAC by 55% in the second pair and by 18% in the third pair.
- In beams with the forced redistribution of bending moments, the degrees of moment redistribution for LWAC beams were lower than those predicted according to that in [39,43], but the difference was much more significant regarding the comparison with the values calculated according to that in [39], which permits the higher value of the degree of moment redistribution without an explicit check on the rotation capacity.

It follows from the above conclusions that normal-weight concrete in statically indeterminate elements cannot be freely replaced with LWAC by only considering compressive strength. When designing reinforced concrete elements made of lightweight aggregate concrete, it is necessary to consider its limited deformation capabilities and lower ductility. The authors recommend that the rotational capacity of the cross-sections in statically indeterminate elements should be checked explicitly in each case.

## Figures and Tables

**Table 1:** Details of beam specimens.

Specimen	Geometry			Longitudinal Reinforcement Details				
	*L_span_*	*h*	*b*	*A_s,top_*	*A_s,top,add_*	*ρ* *_s,top + top,add_*	*A_s,bottom_*	*ρ* *_s,bottom_*
	mm	mm	mm	-	-	-	-	-
BL1	2350	400	250	2#16	2#12	0.0069	2#16	0.0044
BL2					-	0.0044		
BL3					2#20	0.0113		
BN1					2#12	0.0088		
BN2					-	0.0044		
BN3					2#20	0.0113		

**Table 2:** Properties of the steel reinforcing bars used.

Diameter	Net Area	Yield Strength *f_y_*	Tensile Strength *f_u_*	Yield Strain *ε_y_*	Ultimate Strain *ε_u_*	Modulus of Elasticity *E_s_*
mm	mm^2^	MPa	MPa	‰	‰	GPa
8	49.3	653.4 (3/5.%) ^1^	733.2 (3/5.5%)	3.12	62.2	209.4 (3/4.2%)
12	112.6	663.0 (3/0.3%)	748.5 (3/0.2%)	3.28	114.5	202.3 (3/0.8%)
16	197.0	624.1 (3/0.2%)	729.8 (3/0.2%)	3.25	100.0	192.0 (3/2.8%)
20	310.5	595.0 (3/0.1%)	694.7 (3/0.2%)	2.99	90.0	198.4 (3/7.0%)

^1^ Values in parentheses describe the number of samples and the corresponding coefficient of variation characterizing the test results.

**Table 3:** Mixture proportions of the LWAC and NWC concrete.

Type	w/c ^1^	Content per 1 m^3^						
		Cement	Ash	Zeolit	Sand 0/2	Certyd 4/9	Gravel 2/8	Water
		kg	kg	kg	kg	kg	kg	kg
LWAC	0.69	270 (15.2%) ^2^	70 (3.9%)	15 (0.85%)	605 (34.1%)	620 (34.9%)	-	185 (10.4%)
NWC	0.66	280 (11.9%)	-	-	800 (34.2%)	-	1070 (45.7%)	185 (7.9%)

^1^ Takes into account admixtures, namely plasticizers and superplasticizers. ^2^ Values in parentheses describe weight fractions.

**Table 4:** Concrete properties on the day of the test.

Specimen		Concrete Properties on the Day of the Test				
	Age	*f_c_/f_lc_*	*f_c,cube_/f_lc,cube_*	*f_ct,sp_/f_lct,sp_*	*ρ*	*E_c_/E_lc_*
	days	MPa	MPa	kg/m^3^	kg/m^3^	GPa
BL1	147	50.0 (3/2.0%) ^1^	57.2 (3/3.8%)	4.25 (3/5.9%)	1840	19.5
BL2	138	50.0 (3/1.6%)	53.4 (3/3.5%)	4.20 (3/2.4%)	1840	19.8
BL3	158	50.0 (3/0.8%)	57.3 (3/4.6%)	4.40 (3/9.1%)	1850	20.6
BN1	152	40.0 (3/1.2%)	51.3 (3/6.4%)	4.60 (3/13.0%)	2280	28.3
BN2	143	42.4 (3/1.2%)	49.6 (3/3.8%)	4.35 (3/6.9%)	2270	29.0
BN3	161	45.0 (3/6.0%)	45.7 (3/3.1%)	3.75 (3/10.7%)	2250	29.4

^1^ Values in parentheses describe the number of samples and the corresponding coefficient of variation, characterizing the test results.

**Table 5:** Summary of test results.

Specimen	At the Initial Flexural Crack ^3^		At the Yielding State		At the Ultimate State	
	*F_cr_*, kN		*F_y_*, kN		Load	Exterior Support Reactions
	Mid-Span ^1^	Internal Support	Mid-Span ^1^	Internal Support	*F_u_*, kN	*R_u_*, kN
BN1	35	50	120.7 ^2^ (~150) ^4^	154.1	166.6	108.7; 113.8
BL1	40	45	151.2	156.5	172.4	119.8; 124.3
BN2	50	50	138.2	118.5	153.0	116.6; 118.7
BL2	40	30	137.5	122.0	152.6	110.8; 111.7
BN3	25	50	129.3	153.4	206.0	117.4; 116.0
BL3	35	45	139.5	~159 ^2^	205.1	121.1; 120.1

^1^ Values recorded for the failed span. ^2^ Result uncertain. ^3^ Values based on visual inspection and Aramis system data. ^4^ Force estimated according to the load-deflection diagram (Figure 12a).

**Table 6:** Strain results.

Specimen	Ultimate Load, *F_u_*	Strain at the Ultimate State					
		Under Exterior Force (Span 1)		Internal Support		Under Exterior Force (Span 2)	
	kN	*ε_s1_*, ‰	*ε_cu_*, ‰	*ε_s1_*, ‰	*ε_cu_*, ‰	*ε_s1_*, ‰	*ε_cu_*, ‰
BN1	166.6	−19.7	2.2	−11.9 (−11.7)	2.5 (2.2)	−31.8	2.4
BL1	172.4	−21.5	4.0	−27.52 (−29.3)	1.2 (3.2)	−28.9	3.5
BN2	153.0	−21.0	2.7	−25.0 (−26.0)	2.7 (2.9)	−34.4	9.0
BL2	152.6	−21.2	1.9	−18.1 (−23.2)	−(3.7)	−23.0	-
BN3	206.0	−36.0	5.4	−31.4	-	−45.2 (−43.4)	4.3 (3.2)
BL3	205.1	−23.6	2.4	−9.0	2.44	−21.0 (−24.2)	2.7 (2.6)

Note: Values recorded with Aramis are given in brackets (200 mm base length was assumed).

**Table 7:** Redistribution results.

Specimen	Load Redistribution Ratio, *η* ^1^		Deflection ^4^		Ductility Ratio, *µ*_Δ_^3^
	Span	Support	Yield State, Δ*_y_*	Ultimate State, Δ*_u_*	
	%		mm		-
BN1	3.44	−7.21	2.91 (4.46) ^2^	12.05	4.14 (2.70) ^2^
BL1	−2.31	4.84	4.01	19.19	4.78
BN2	−11.18	23.45	3.84	19.32	5.03
BL2	−5.40	11.33	4.23	12.74	3.01
BN3	18.09	−37.98	3.39	19.34	5.71
BL3	14.92	−31.27	3.42	13.34	3.90

^1^ Negative value indicates that there was an increase of the moment value compared to the elastic analysis. ^2^ A value for the estimated yield load is given in brackets; see note 4 in Table 5. ^3^ Values were calculated for the mean values of deflection. ^4^ The mean value for both spans.

**Table 8:** Load-bearing capacity.

Specimen	Experimental Load	Calculations According to EC2			Comparison
	*F_u_*, kN	*M_Rd_^+^,* kNm	*M_Rd_^−^,* kNm	*F_uEC2_,* kN	*F_u_/F_uEC2_*, -
BN1	166.6	86.7	136.4	170.0	0.98
BL1	172.4	87.8	137.8	172.1	1.00
BN2	153.0	87.0	87.0	149.8	1.02
BL2	152.6	87.8	87.8	151.2	1.01
BN3	206.0	87.3	207.8	200.6	1.03
BL3	205.1	87.8	208.9	201.7	1.02

## Data Availability

The data presented in this study are available upon request from the corresponding author.
